# Endoscopic approach for the management of a gastrogastric fistula, eroded band, and outlet stenosis after a vertical banded gastroplasty

**DOI:** 10.1016/j.vgie.2024.04.001

**Published:** 2024-04-20

**Authors:** Patricia Ruiz-Cota, Manoel Galvao, Elias Ortiz-Gomez, Stephany M. Márquez-González, Josefina Principe, Santiago Horgan

**Affiliations:** 1 Bariatric Surgery Department, Elias Ortiz & Company Mexico Weight Loss Specialists, Tijuana, Mexico; 2 Division of Minimally Invasive Surgery, Department of Surgery, University of California San Diego, San Diego, California

## Background

Vertical banded gastroplasty (VBG) was a purely restrictive procedure described initially by Manson in 1980.^1^ Although this procedure gained popularity and was commonly performed between the 1980s and early 2000s, it has since been abandoned.^2^, ^3^, ^4^ The initial short-term outcomes of VBG appeared very promising, becoming acceptable and comparable with other restrictive procedures performed at that time.^5^ However, long-term studies have shown high rates of adverse effects, revisional surgery, and weight regain.^6^ The VBG procedure consisted of creating a gastric window at a distance from the gastroesophageal junction and then creating a pouch by dividing the fundus with a non-cutting stapler fired toward the angle of His and encircling the outlet of the lesser curvature with a silastic band or mesh.^5^^,^^7^ Adverse effects related to this procedure consist primarily of staple-line disruption causing gastrogastric fistulas, pouch enlargement, gastric outlet stenosis or stricture, erosion of the band, severe reflux and regurgitation, and vomiting or food intolerance.^3^ Endoscopic approaches have effectively been used to manage adverse effects of bariatric surgery.^8^, ^9^, ^10^ Here, we present a case of endoscopic management of long-term adverse effects after VBG.

## Case presentation

A foreign 50-year-old female patient with a history of laparoscopic VBG for morbid obesity (body mass index = 52 kg/m^2^), performed 20 years earlier (2002), presented to our clinic in Tijuana, Mexico, with long-standing acid reflux and weight regain. She exhibited acceptable weight loss results in the first year after surgery, losing approximately 23 kg, then gradually regained all the lost weight and developed acid reflux, which had been treated with proton-pump inhibitors for the previous 3 years with inadequate symptom control. An EGD was performed due to exacerbation of reflux symptoms (July 2022), which documented a “deformity in the gastric fundus,” a “blind gastric pouch,” and absence of esophagitis. This led us to discuss various approaches with the patient. Due to her high surgical risk, the endoscopic procedure was preferred as the best option (December 2022).

## Procedure

**Figure 1:**
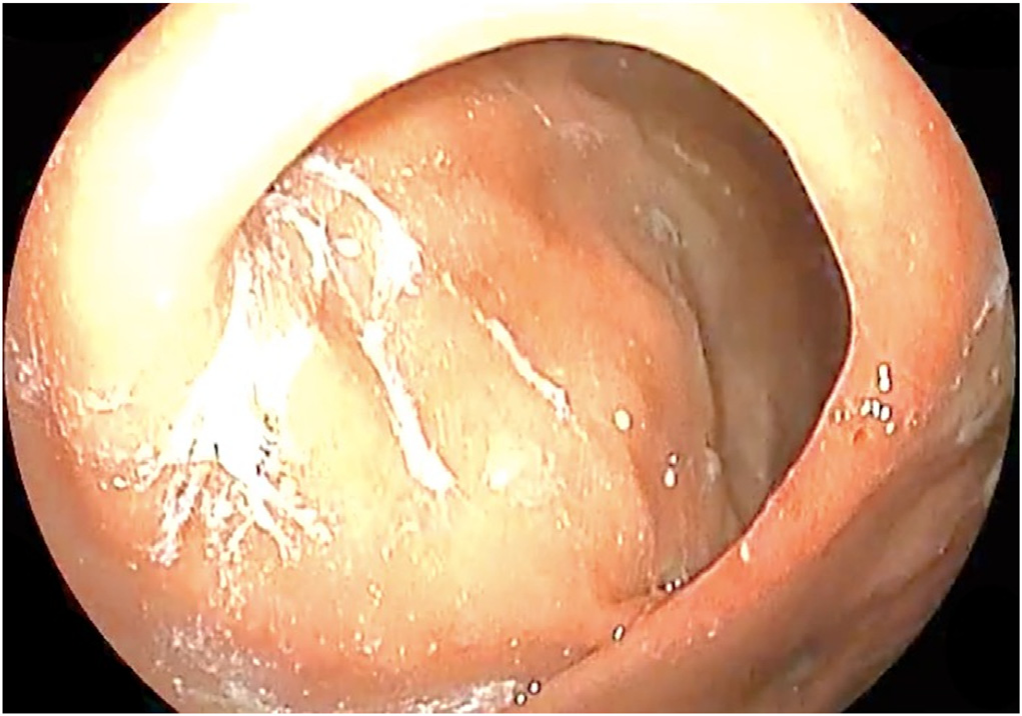
Gastro-gastric fistula 2 cm from the gastroesophageal junction.

**Figure 2:**
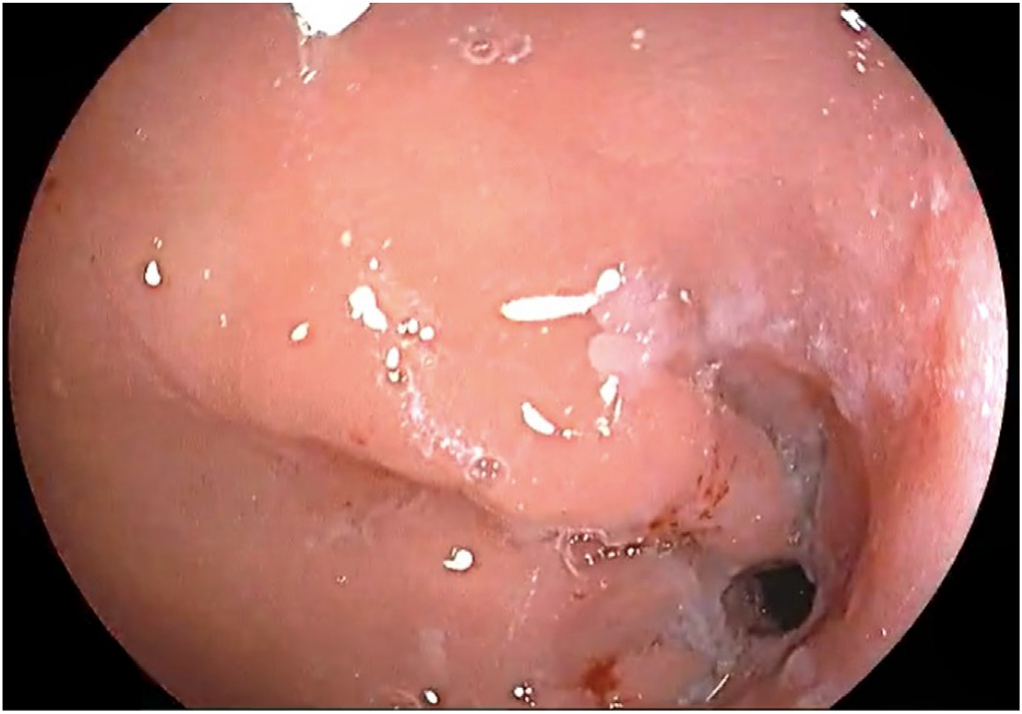
Gastric outlet stenosis of 1 cm, distal to the pouch.

**Figure 3:**
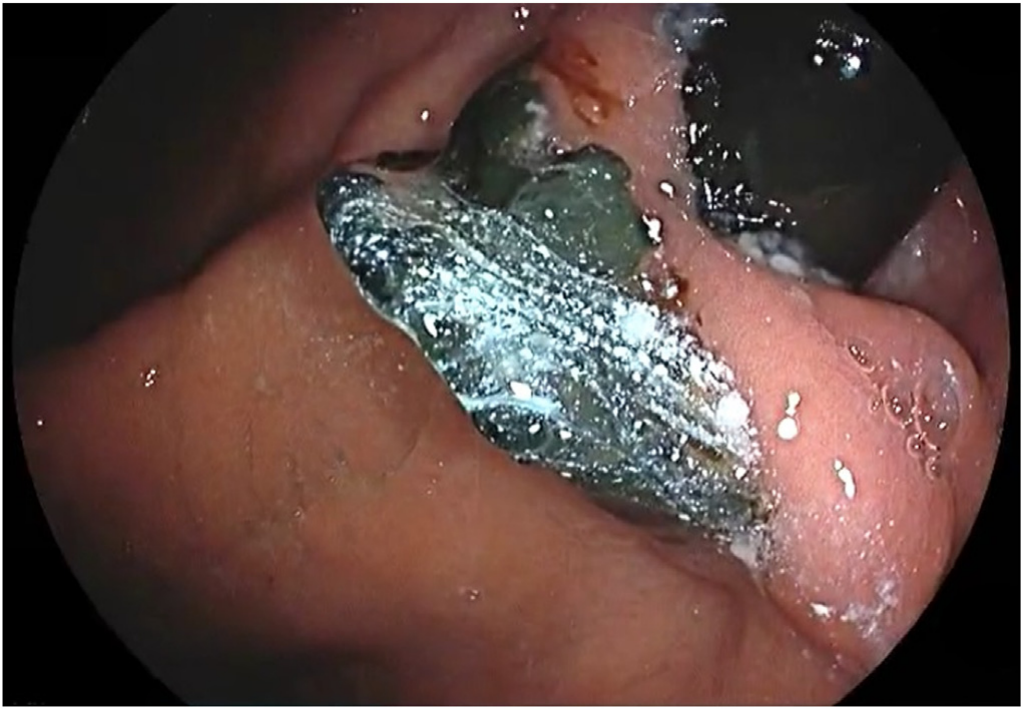
Eroded band in the gastric body.

**Figure 4:**
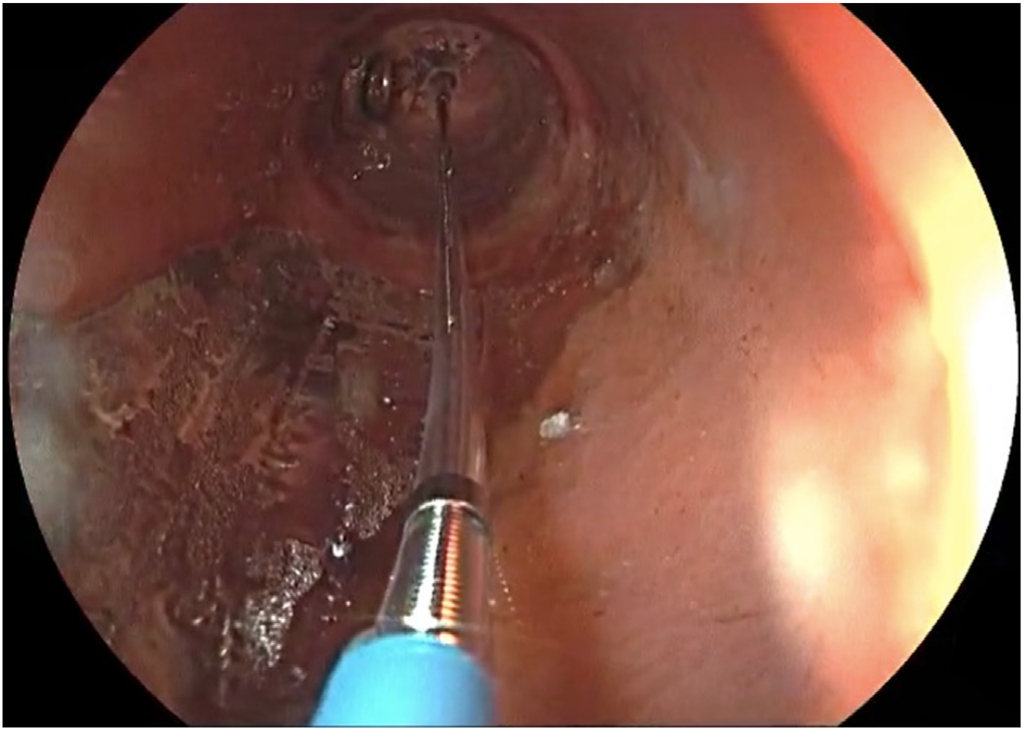
Dilatation of the gastric outlet stenosis with 20-mm hydrostatic balloon dilator.

The patient was placed in a left lateral recumbent position under general anesthesia. A repeat diagnostic EGD was then performed with the Fujifilm ELUXEO 7000 video endoscopy system (Video 1, available online at www.videogie.org), revealing the following: a gastrogastric fistula 2 cm from the esophagogastric junction (Fig. 1), a 14-cm tubular pouch with 1-cm distal stenosis (Fig. 2), a silastic band thoroughly eroded into the lumen of the gastric body (Fig. 3), and absence of esophagitis. With the EI-740D/S dual-channel endoscope, the gastric outlet stenosis was dilated with a 20-mm hydrostatic balloon dilator (Fig. 4). Subsequently with endoscopic scissors, the eroded band was carefully cut, detached, and removed in small pieces transorally from the gastric body.

**Figure 5:**
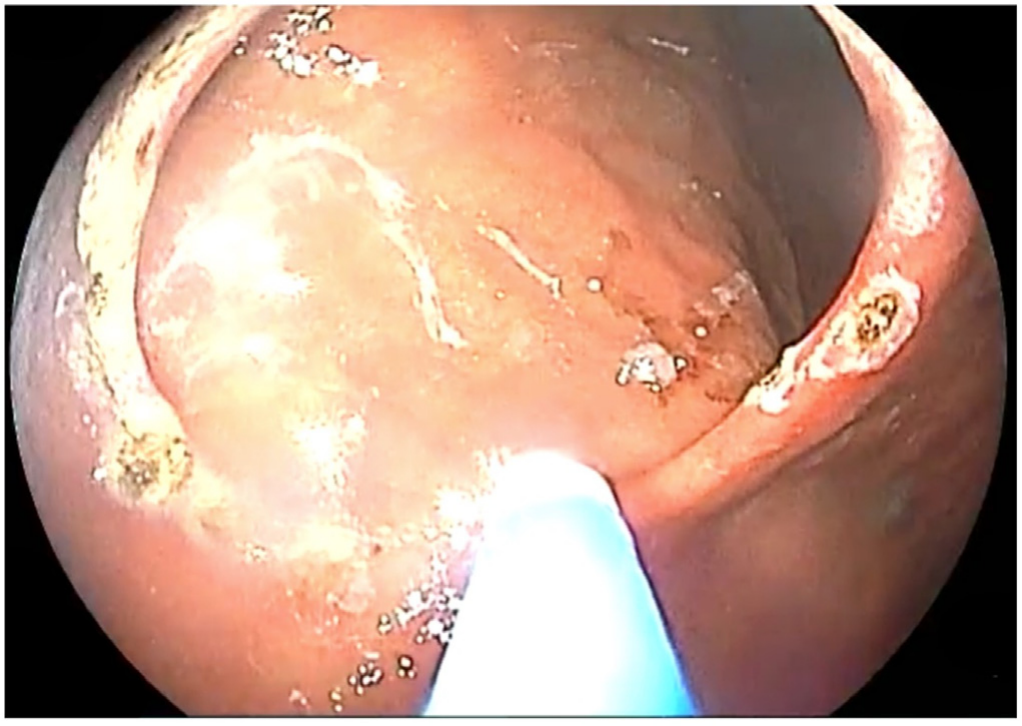
Electrofulguration of the edges of the gastro-gastric fistula with argon plasma.

**Figure 6:**
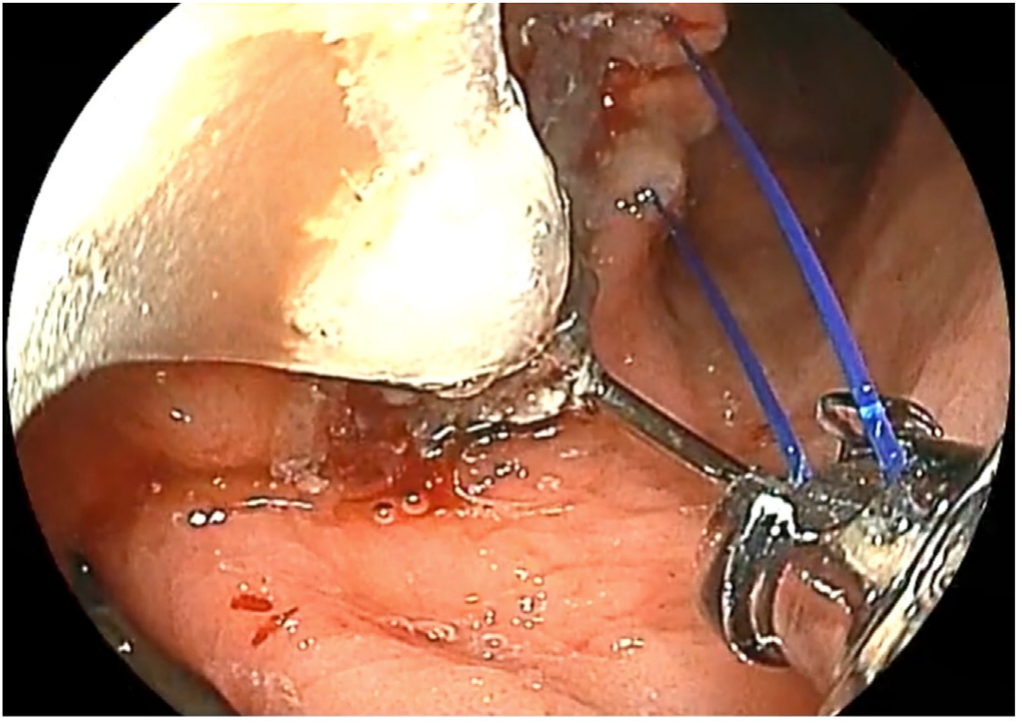
Closure of the gastro-gastric fistula with the “two single running sutures” technique using the Apollo OverStitch Endoscopic Suturing System (Apollo Endosurgery, Austin, Tex, USA).

**Figure 7:**
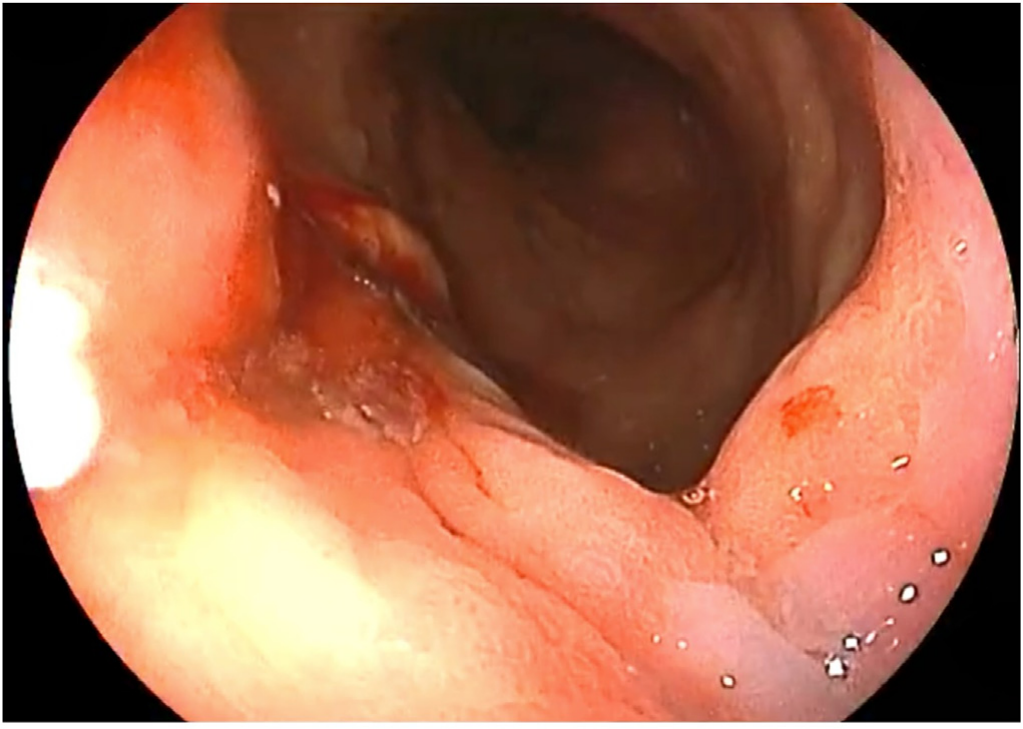
Complete closure of the gastro-gastric fistula was achieved, with recreation of the gastric pouch.

To repair the gastrogastric fistula, first electrofulguration was performed on the edges of the fistula, following the annular shape of the fistula, with argon plasma coagulation (Fig. 5). The closure was reinforced with the “two single running sutures” technique using the Apollo OverStitch Endoscopic Suturing System (Apollo Endosurgery, Austin, Tex, USA) (Fig. 6). Complete closure of the fistula was achieved, with recreation of the gastric pouch (Fig. 7).

**Figure 8:**
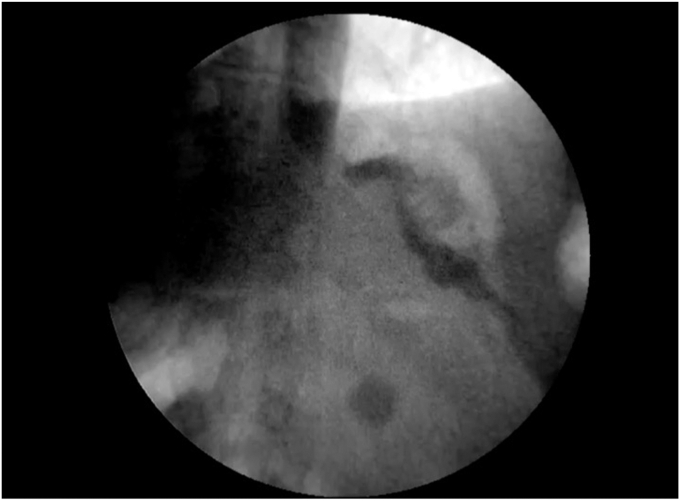
Fluoroscopic imaging revealed no passage of contrast to the gastro-gastric fistula repair, adequate flow through the dilatation, and no extraluminal effusion.

On day 1 post-operation, a fluoroscopy was performed, demonstrating integrity of the sutures with no passage of contrast through gastric fundus, an adequate flow through the gastric dilatation, and no extraluminal effusion (Fig. 8). The patient presented an uneventful recovery and was discharged on day 2 post-operation with resumption of oral feeding. The patient returned to her city of origin. During follow-up, which was conducted remotely, we emphasized the importance of imaging studies to ensure sustained fistula closure. The patient reported no acid reflux symptoms after 3 and 6 months of follow-up. Additionally, body mass index decreased to 48.6 kg/m^2^ and 39 kg/m^2^, respectively.

## Conclusion

Endoscopic approaches should be the first line of treatment for adverse effects associated with VBG. Given that VBG was widely performed years ago, we will likely continue to encounter these types of adverse effects in our medical practice. As presented here, endoscopic management offers a suitable, safe, and effective intervention.

## Disclosure

Dr Galvao is a scientific advisor for Apollo EndoSurgery and Keyron; consultant for GI Dynamics, Apollo EndoSurgery, USGI, Colubris Mx, Scitech, and MITech; and speaker for Olympus LA, Erbe, and Medtronics LA. Dr Horgan is a consultant for Stryker Corporation, Intuitive Surgical, Fortimedix Surgical, and Alume Biosciences. All other authors disclosed no financial relationships relevant to this publication.
